# High-Dose Fluorescein Reveals Unusual Confocal Endomicroscope Imaging of Low-Grade Glioma

**DOI:** 10.3389/fneur.2021.668656

**Published:** 2021-07-16

**Authors:** Evgenii Belykh, Naomi R. Onaka, Xiaochun Zhao, Irakliy Abramov, Jennifer M. Eschbacher, Peter Nakaji, Mark C. Preul

**Affiliations:** ^1^Department of Neurosurgery, The Loyal and Edith Davis Neurosurgical Research Laboratory, St. Joseph's Hospital and Medical Center, Barrow Neurological Institute, Phoenix, AZ, United States; ^2^Department of Neuropathology, Barrow Neurological Institute, St. Joseph's Hospital and Medical Center, Phoenix, AZ, United States

**Keywords:** confocal laser endomicroscopy, fluorescein sodium, fluorescence-guided surgery, low-grade glioma, nonenhancing glioma, oligodendroglioma

## Abstract

**Background:** Fluorescence-guided brain tumor surgery using fluorescein sodium (FNa) for contrast is effective in high-grade gliomas. However, the effectiveness of this technique for visualizing noncontrast-enhancing and low-grade gliomas is unknown. This report is the first documented case of the concurrent use of wide-field fluorescence-guided surgery and confocal laser endomicroscopy (CLE) with high-dose FNa (40 mg/kg) for intraoperative visualization of tumor tissue cellularity in a nonenhancing glioma.

**Case Description:** A patient underwent fluorescence-guided surgery for a left frontal lobe mass without contrast enhancement on magnetic resonance imaging. The patient received 40 mg/kg FNa intravenously at the induction of anesthesia. Surgery was performed under visualization with a Yellow 560 filter and white-light wide-field imaging. Intraoperative CLE produced high-quality images of the lesion 1.5 h after FNa injection. Frozen-section analysis demonstrated findings comparable to those of intraoperative CLE visualization and consistent with World Health Organization (WHO) glioma grades II–III. The patient recovered without complications. Analysis of the permanent histologic sections identified the tumor as an anaplastic oligodendroglioma, IDH-mutant, 1p/19q co-deleted, consistent with WHO grade III because of discrete foci of hypercellularity and increased mitotic figures, but large regions of the lesion were low grade.

**Conclusions:** The use of high-dose FNa in this patient with a nonenhancing borderline low-grade/high-grade glioma produced actionable wide-field fluorescence imaging using the operating microscope and improved CLE visualization of tumor cellularity. Higher doses of FNa for intraoperative CLE imaging and possible simultaneous wide-field fluorescence surgical guidance in nonenhancing gliomas merit further investigation.

## Introduction

Fluorescein sodium (FNa) is a fluorescent biomarker available as a water-soluble dye that has been widely used in ophthalmology since the early 1960s. Moore et al. (1) reported anecdotal clinical use of fluorescein to help localize intracranial neoplasms as early as 1948. Although FNa has demonstrated utility in increasing the extent of resection in high-grade gliomas (HGG), its efficacy has not been well-elucidated in low-grade gliomas (LGG), and there is currently a paucity of investigation on what FNa-guided resection may reveal when used for LGG (2–5). In a systematic review of fluorescence-guided glioma surgery, Senders et al. (6) identified 11 studies describing the use of FNa in glioma resection. All 11 studies included patients with HGG, whereas only three studies included patients with LGG.

FNa is commonly used for retinal angiographic procedures, and it has been shown to have few side effects with oral ingestion; the most common adverse effects are nausea and vomiting (7, 8). Additional adverse effects of intravenous FNa include postoperative yellow discoloration of urine at lower doses, such as 3–4 mg/kg, and skin or sclera discoloration at higher doses; nonetheless, overall, it is confirmed to be safe for patients (9). Only two cases of anaphylactic reactions have been reported after neurosurgical administration of FNa, both with an FNa dose of 20 mg/kg (10, 11).

FNa is useful in identifying intracranial malignancies because it extravasates from cerebral vessels in places where the blood-brain barrier has been damaged. This mechanism allows the dye to concentrate, localizing the area where tumor invasion has disrupted vascular integrity. The pattern of FNa extravasation is relatively unique in HGG compared to that in simple surgical trauma to brain tissue, and the pattern indicates a disruption in the blood-brain barrier (12). FNa is excited by light in 460–500 nm wavelengths, and it emits radiation in the 540–690 nm range. Light filters, such as the Yellow 560 filter (Carl Zeiss Meditec AG, Oberkochen, Germany), on surgical microscopes help to visualize the fluorescing tissue. However, visualization and surgical maneuvering during resection often require switching between the filter and wide-field white-light illumination. The range of FNa doses used for neurosurgical application has varied and has been subjectively divided into a “low-dose” (1–10 mg/kg) range that requires a fluorescence detection module on a neurosurgical operating microscope for identification and a “high-dose” (15–20 mg/kg or greater) range in which the naked eye can detect the fluorescent staining even without a fluorescence detection module (13).

In conjunction with dedicated fluorescein filters on surgical microscopes, the increasing use of intraoperative histopathologic examination with confocal laser endomicroscopy (CLE) using FNa has produced a tool for optically interrogating gliomas with a sensitivity and specificity comparable to the examination of a frozen section (14–18). Our institutional experience with CLE and low-dose FNa has been similar to that of others, albeit inconsistent for nonenhancing lesions and LGG. However, in this case report, we describe a situation in which high-dose FNa was administered during resection of an oligodendroglioma with the use of CLE, which produced unusually clear images of the LGG. This report contributes to the limited evidence for the possible use of FNa to visualize LGG, particularly with the aid of CLE. The CLE images indicate that a relatively high dose of FNa (e.g., 20–40 mg/kg) may produce a significant benefit in identifying tumors heretofore not labeled well with lower doses of FNa during fluorescence-guided surgery.

## Case Description

**Figure 1:**
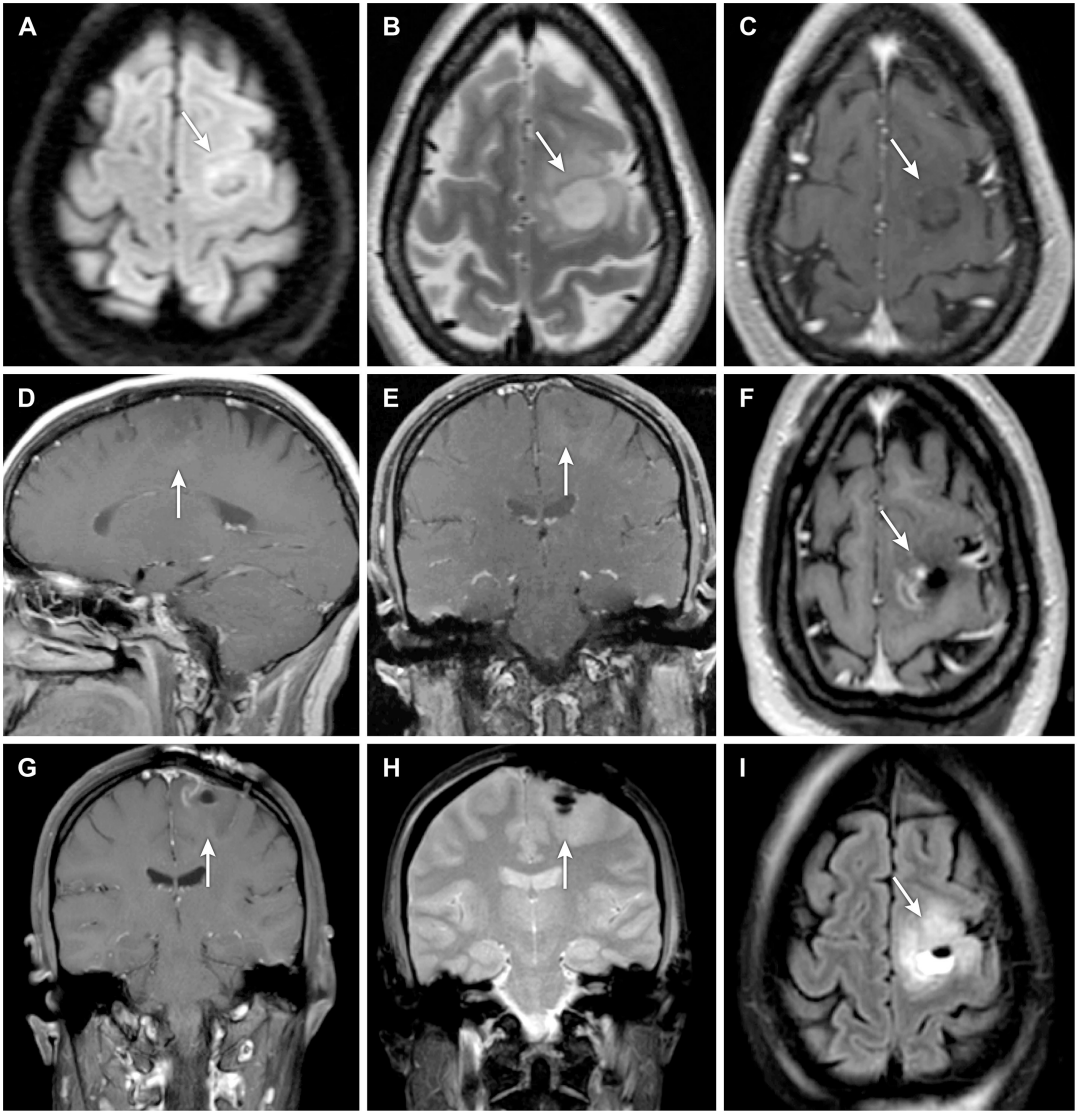
**(A–E)** Preoperative and **(F–I)** postoperative magnetic resonance imaging (MRI) of a patient with a nonenhancing lesion (**A–I**, *arrows*) in the left precentral gyrus. Preoperative MRIs shown are **(A)** axial diffusion-weighted, **(B)** axial T2-weighted, **(C)** axial T1-weighted, **(D)** sagittal T1-weighted with contrast, and **(E)** coronal T1-weighted with contrast. Postoperative MRIs shown are **(F)** axial T1-weighted with contrast, **(G)** coronal T1-weighted with contrast, **(H)** coronal diffusion-weighted, and **(I)** axial diffusion-weighted. Used with permission from Barrow Neurological Institute, Phoenix, Arizona.

**Figure 2:**
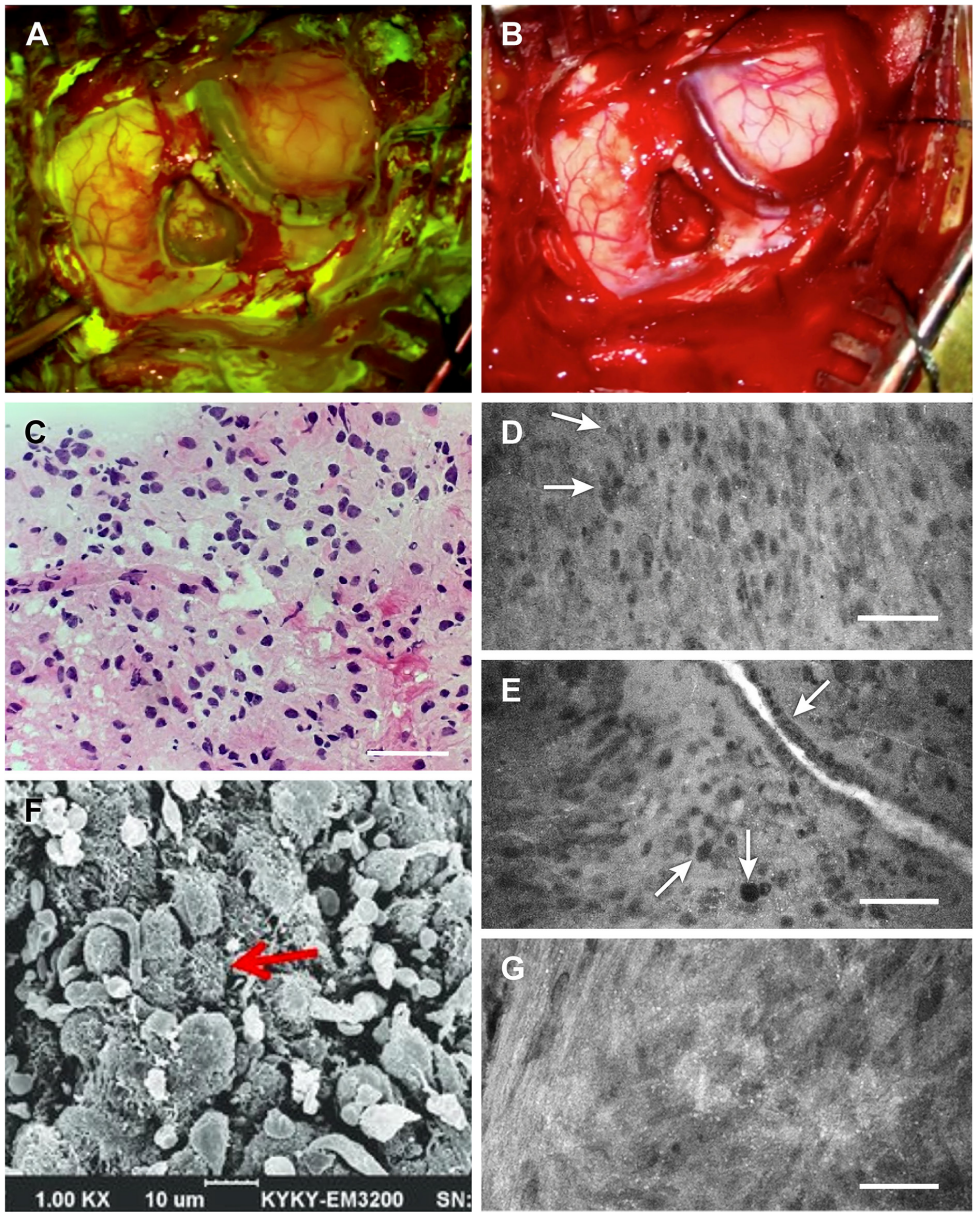
Intraoperative tumor visualization. Exposure during tumor resection with **(A)** Yellow 560 filter and **(B)** standard white light. **(C)** Photograph of intraoperative frozen-tissue section slide interpreted as grade II glioma. **(D)** Confocal laser endomicroscopy (CLE) image showing subtle discrete cellular aggregates (*arrows*) within a relatively widespread area of cells interpreted as consistent with lower-grade glioma 1.5 h after injection of 40 mg/kg fluorescein sodium (FNa). **(E)** A vessel 1.5 h after injection of 40 mg/kg FNa showing irregular endothelium (*top arrow*), with a focus of cells indicating pleomorphism relative to surrounding cells (*center arrow*) and what appears to be a mitotic figure (*bottom arrow*). **(F)** Scanning electron microscopy (SEM) image showing glioma cell morphology with multiple small vesicles and protrusions. **(G)** CLE image of another tumor area demonstrates small bright dots that likely represent cell surface vesicles with fluorescein, which corresponds well to the SEM microscopy of the cells. These bright dots were visible throughout the tumor on CLE images. Scale 50 μm **(D,E,G)**. Panels **(A–E,G)** are used with permission from Barrow Neurological Institute, Phoenix, Arizona. Panel **(F)** is adapted with permission from Lv et al. (22), CC-BY 4.0.

A patient with a 4-year history of headaches was admitted for new-onset generalized seizure. Magnetic resonance imaging (MRI) showed a nonenhancing 1.6 × 1.3-cm heterogeneous posterior left frontal lobe mass just anterior to the precentral gyrus, with a surrounding abnormal high signal on T2-weighted and fluid-attenuated inversion recovery (FLAIR) imaging, interpreted as probably representing edema (Figure 1). The most likely differential diagnoses considered were metastatic disease and primary brain lesion. The patient gave voluntary informed consent to participate in the CLE imaging study, which was approved by the St. Joseph's Hospital and Medical Center Institutional Review Board (No. 10BN130). In the operating room, FNa was administered to the patient shortly after the induction of anesthesia. However, instead of the typical dose of 2–5 mg/kg, a higher dose of 40 mg/kg was administered. This higher dose was within the acceptable dosing range for FNa. When the tumor area was mapped for motor function, both the posterior and anterior regions immediately adjacent to the tumor showed some motor function. Yellow discoloration of the tissue was observed because of the higher FNa dose (Figures 2A,B). The dye concentrated in the area of the tumor, which allowed visualization of its borders, and the tumor was resected completely.

**Figure 3:**
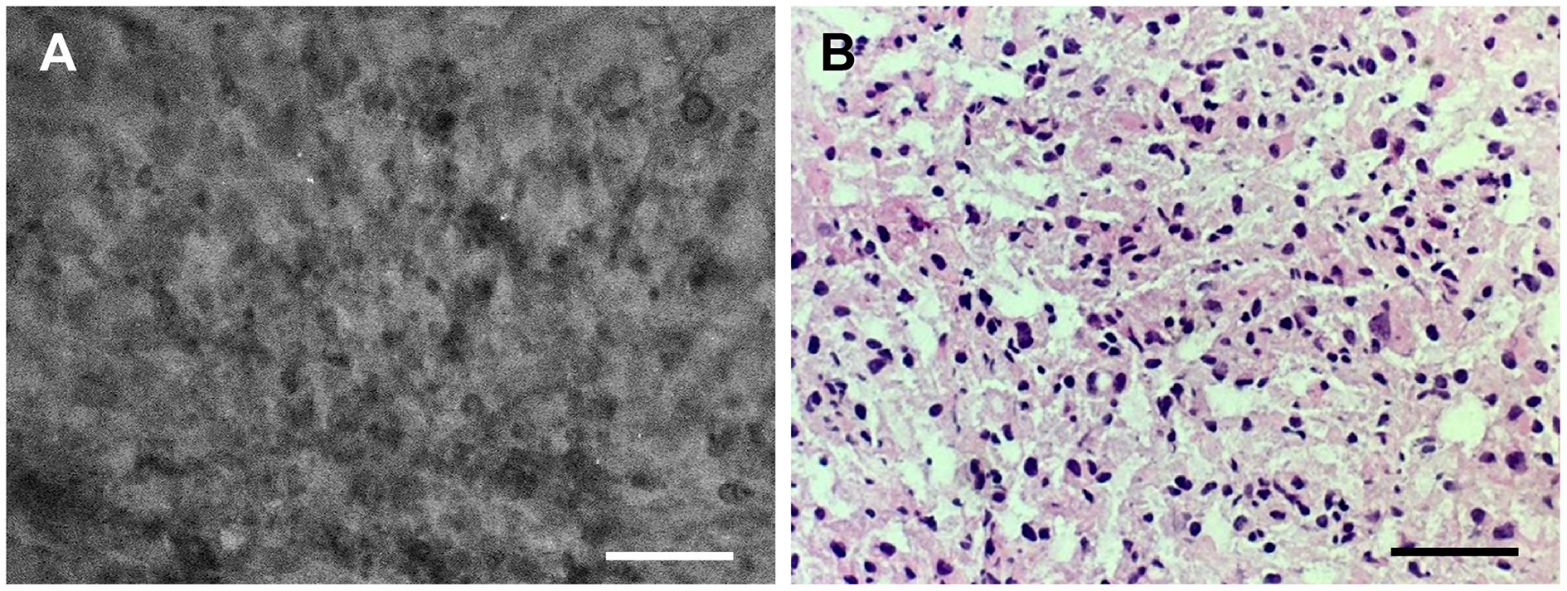
Good correlation of the **(A)** confocal laser endomicroscopy image collocated with the **(B)** hematoxylin-eosin–stained frozen section interpreted as grade II glioma. Scale bars, 50 μm. Used with permission from Barrow Neurological Institute, Phoenix, Arizona.

Four biopsies from within the tumor region (the last obtained about 1.5 h after FNa administration) were subjected to immediate intraoperative *ex vivo* CLE imaging (Convivo, Carl Zeiss Meditec AG). CLE imaging demonstrated hypercellular brain architecture, with abnormal cells suggestive of a cellular tumor in all biopsy specimens (Figures 2C–G). Histologic findings on frozen sections were consistent with glioma grades II–III, with the wide area majority characteristic of grade II glioma (Figure 3). Further histologic assessment of the permanent sections revealed variable cellular and infiltrative glioma with rounded oligodendroglial morphology and prominent perinuclear halos. The tumor was assigned the overall classification of anaplastic oligodendroglioma, isocitrate dehydrogenase (IDH)-mutant, 1p/19q codeleted, with ATRX expression because of the regions of more aggressive heterogeneity (discrete foci of hypercellularity and increased mitotic figures) but also extensive areas of lower-grade tumor.

**Figure 4:**
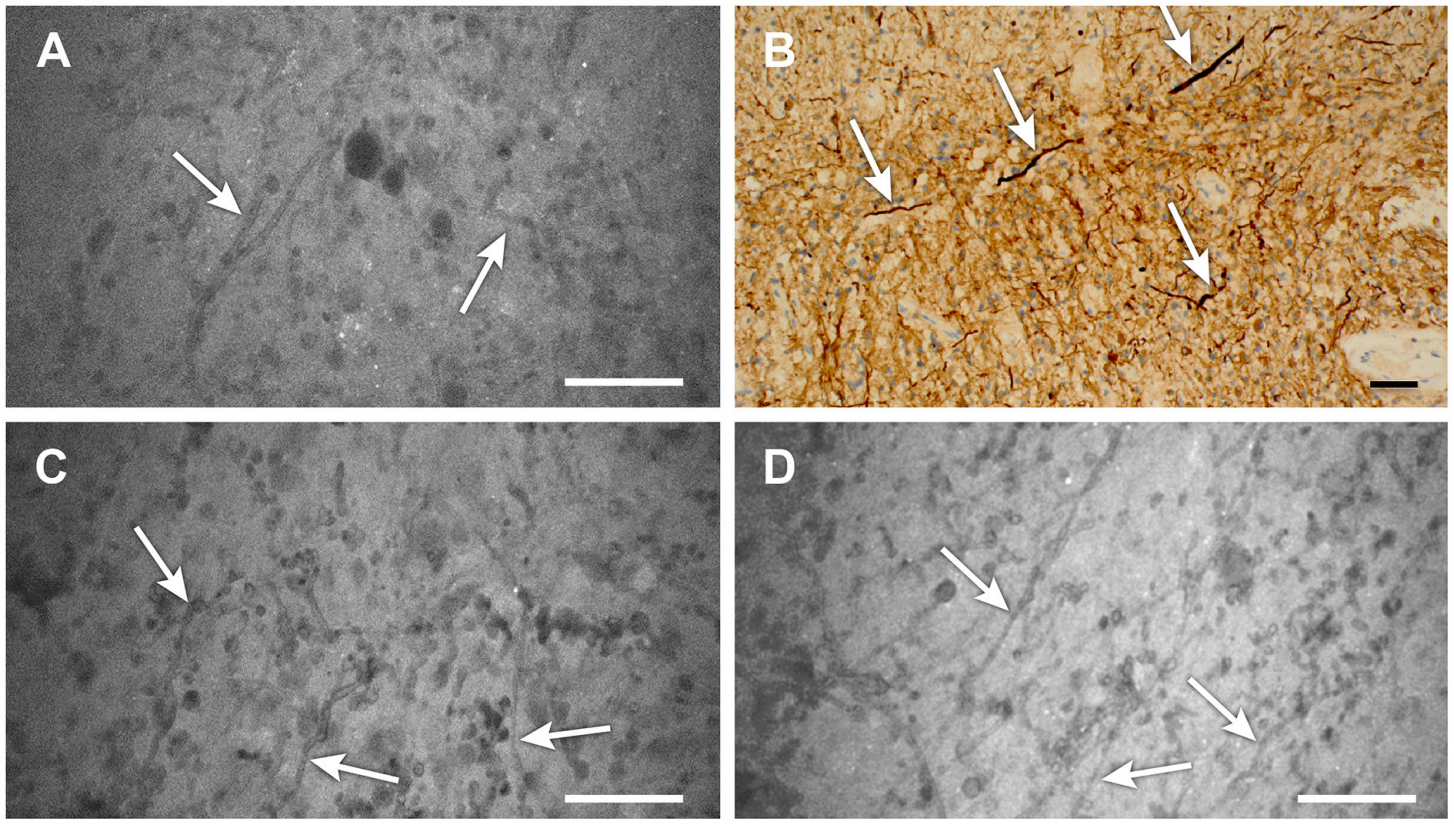
**(A–D)** Confocal laser endomicroscopy (CLE) images showing areas of low-grade glioma **(A,C,D**) that reveal distinct strand-like structures or fibers throughout the tumor (scale 50 μm). **(B)** Image from the same area stained for neurofilaments (*arrows*) also shows these structures, which are likely swollen axons resulting from tumor edema (scale bar, 20 μm). We have not previously observed these detailed structures on CLE images with low-dose fluorescein sodium (FNa) staining. Their notable appearance here is likely related to the high dose of FNa used in this case. Used with permission from Barrow Neurological Institute, Phoenix, Arizona.

CLE concurrent with high-dose FNa provided extremely clear images of cellular architecture, mitotic figures, endothelium of vessels, and swollen axons (Figure 4). The brightness and clarity of the CLE images revealed a distinct morphologic appearance not typically observed with lower-dose FNa, especially 1.5 h or longer after administration (but often in even less time). The patient tolerated this dose well, and the patient's postoperative yellowish skin discoloration resolved rapidly.

## Discussion

Gliomas are classified according to WHO grades I–IV, with LGG encompassing grades I and II. Still, they are representative of a heterogeneous group of tumors with wide variability in terms of prognosis and treatment. In the case description, we present the immunohistochemistry and pathologic characteristics identifying the tumor as an anaplastic oligodendroglioma, IDH-mutant, 1p/19q codeleted, with ATRX expression isolated within a vast majority of areas that were lower-grade glioma. Although the tumor was classified as a grade III glioma because of regions of increased aggressive characteristics, the prognostic significance of grade II vs. III in this tumor class compared with that of a nonanaplastic oligodendroglioma with an otherwise similar profile is not clearly understood, with the diagnostic criteria for histopathologic grading introducing subjectivity in interobserver variability in grading these lesions (19).

The tumor in this patient was categorized as grade II rather than grade III in the initial frozen-section analysis. It was noncontrast enhancing, which is a characteristic most often observed with LGG. The vast majority of this tumor was of a low-grade II glioma. The tumor was assigned the grade III classification because some localized regions were interpreted to be more aggressive. Low-grade tumors are often show localized areas or have heterogeneous regions in transition to more aggressive states.

Despite the grade III classification, our findings support the utility of high-dose FNa in the resection of LGG because the imaging correlated to the histology within the regions of lower-grade tumor.

The higher dose of 40 mg/kg FNa administered to the patient revealed fine intraoperative imaging characterization using CLE for areas of hypercellularity and tissue. The higher than usual dose of FNa produced unusually excellent contrast for the CLE visualization of the tumor. In conjunction with earlier studies commenting on the potential use of FNa in the resection of LGG, our report highlights several areas of interest, including the optimal dose of FNa and which grades and characteristics of LGG may be most amenable for FNa-guided resection.

### CLE With Low-Dose FNa for Low-Grade Glioma

Our previous experience with CLE for LGG demonstrated that FNa contrast was usually inadequate for clear visualization of histologic characteristics, especially 1 h or longer after administration of FNa (15). In each patient, 5 mg/kg FNa was injected, and intraoperative CLE was performed within a few minutes after its administration. In 66 patients, 8 grade II gliomas were identified, 10 grade III, and 3 grade IV. The exact histopathologic diagnosis of the tumors was not possible for every biopsy or every patient because of the lack of image contrast and clarity to view mitoses adequately enough to count them reliably.

Chen et al. (5) found that, in four gross total resections of LGG, three gliomas were clearly stained after injection of high-dose FNa (15–20 mg/kg). In preoperative MRIs, these three tumors also showed enhancement, which is generally found in higher tumor grades. However, in one patient whose LGG was nonenhancing on preoperative MRI, the staining was not clear. Notably, FNa still provided utility in this case because the surgeons could resect around nebulously stained areas, prompting Chen et al. (5) to conclude that FNa may be useful in treating LGG, although this aspect was not specifically evaluated in their investigation.

Schebesch et al. (4) reported five patients with nonenhancing preoperative MRIs but gliomas positive for FET-PET (^18^F-fluoroethyl-l-tyrosine–positron emission tomography), which they referred to as nonenhancing gliomas. All five patients received 5 mg/kg FNa, and the Yellow 560 filter was used as needed. These investigators found that, despite the lack of contrast enhancement on MRI in all patients, all five tumors demonstrated some degree of fluorescence, which correlated with PET metabolic activity. One grade II tumor was visible only under the fluorescence-detecting filter. Final grading identified two LGG and three HGG as grade III. Nonetheless, Schebesch et al. (4) concluded that the incorporation of FNa was helpful both in detecting lesions and in ascertaining their borders, regardless of grade.

Notably, in the report by Schebesch et al. (4), abnormalities were found in the FLAIR and T2 sequences of preoperative MRIs in all five patients in their series. These abnormalities may be sensitive markers of a damaged blood-brain barrier, and they may be present before the tumor becomes contrast-enhancing with progression. In the patient detailed in our case report, preoperative imaging showed similar findings in that the tumor was noncontrast enhancing, but there was a highly abnormal T2/FLAIR signal surrounding the mass. Ultimately, two of the five patients in the Schebesch et al. (4) report were found to have grade III anaplastic oligodendrogliomas, which was the same diagnosis as in our patient. Further studies using FNa may permit elaboration of the characteristics of each tumor subtype under visualization with FNa, and our findings in combination with those previously reported by other authors compel further exploration in future studies.

### CLE With High-Dose FNa for Low-Grade Glioma

Extrapolating from the existing literature and from our observations as detailed in this report, we believe that the application of FNa is influenced by its dosing, both in terms of visualization with or without the Yellow 560 filter and with its beneficial administration in patients with lower grades of glioma. In a study of FNa-guided resection of glioblastoma, Shinoda et al. (20) found that high doses of FNa (20 mg/kg) produced usable fluorescence that was viewable under white-light illumination, without the need for an operating microscope Yellow 560 filter. Their report supports the evaluation that higher doses of FNa can better identify the tumor area and borders. However, their results were obtained from imaging of high-grade lesions with an ostensibly larger degree of blood-brain barrier disruption. Thus, the results may not be translatable to gliomas demonstrating nonenhancement on MRIs. Unlike 5-aminolevulinic acid (5-ALA), which does not usually demonstrate cellular uptake in low-grade tumors, high-dose FNa may provide fluorescent visualization and discrimination of the cellular architecture and tumor margin zone.

The question of whether to use FNa when dealing with either low-grade or high-grade tumors is thus germane for wide-field operative microscopy and CLE imaging. Currently, the intraoperative redosing of FNa is used as an off-label FNa application in the United States. In most cases, FNa is administered at the beginning of the surgery (21), as we have performed it early on. However, exposure of the tumor and the beginning of resection, as well as later inspection of surgical margins, may require many minutes to hours before the use of imaging such as CLE. In our experience, we have begun to delay FNa administration until after anesthesia induction but immediately before surgical work begins on the tumor, which allows for brighter and clearer images on CLE imaging. However, the timing of FNa administration is a judgment call because later administration of FNa after accessing the tumor or beginning its surgical resection may highlight surgically damaged tissue as viewed with wide-field imaging, making it almost useless for delineating tumor margin. In most instances, the FNa signal on wide-field imaging is confined to the tumor before it progressively saturates surrounding tissue. This characteristic of FNa can be confusing or disconcerting on wide-field imaging because of the fluorescence of nonrelevant tissues, areas of bleeding, or areas with disruption of tissue during the normal process of surgery.

In many cases where 2–5 mg/kg FNa was administered early in the procedure, we acquired CLE imaging that was suboptimal, uninterpretable, or too dark. Thus, we have redosed the FNa to acquire improved CLE images (16). In the current case report, the single 40 mg/kg FNa dose produced a signal that remained bright and produced excellent CLE images even 1.5 h after administration. This case is fortuitous in that it may show implications for very-high-dose FNa administration, where in the past, FNa fluorescence has not been particularly delineative of LGG when used with wide-field operative microscopy.

The two cases where anaphylactic reactions occurred minutes after FNa injection showed decreased blood pressure, bradycardia or tachycardia, and flushing over the infusion anatomical area (10, 11). These patients responded to adrenaline, atropine, prednisolone, and dopamine administration. Surgeries were halted, and the patients were moved to the intensive care unit. In both cases, elevated laboratory values of tryptase were found, while in one case, the IgE value was also increased. In both instances, the patients fully recovered, and one report cited the occurrence of such a reaction for the first time in 121 patients injected with FNa for neurosurgery (10).

When the goal is to produce actionable fluorescence image surgical guidance with a wide-field fluorescence detection system, especially when using CLE, a higher dose of FNa may be of more benefit if only one dose is administered at the beginning of an operation. This approach may be particularly useful when using sensitive imaging such as CLE for discriminating the histoarchitecture of tumor margins, especially for LGG tissue that may not be as amenable to 5-ALA fluorescence guidance. Simultaneous use of fluorophores such as 5-ALA and FNa may be an option for low-grade tumors or nonenhancing tumors. Although 5-ALA has not been shown to delineate low-grade tumors well, isolated regions of low-grade gliomas often transition to a more metabolically aggressive type. The use of 5-ALA may discriminate those isolated regions, whereas high-dose FNa could help define the general tumor area of low-grade tissue and margins. Of course, the logistics of administering the different fluorophores for optimal uptake by tumor cells should be considered. FNa dose-escalation studies in LGG may be useful to define the minimal dosage that allows better interpretation of CLE images to delineate the histologic tumor features and, importantly, the tumor margins or invaded regions. CLE is currently in its infancy regarding *in vivo* surgical application. Cases such as ours may indicate a need for expanded or explorative fluorescence techniques.

## Conclusions

This case report demonstrates the excellent quality available with dual-fluorescence visualization (wide-field operative microscope Yellow 560 fluorescence navigation and CLE imaging) with a high dose of FNa in a patient with a nonenhancing glioma up to 1.5 h after FNa administration. This report supports the investigation of higher doses of FNa in nonenhancing gliomas and gliomas suspected to be a lower grade that usually do not exhibit 5-ALA-mediated fluorescence. Given the highly informative images, the minimal adverse effects of FNa, and the good postoperative outcome of this case, higher doses of FNa (20–40 mg/kg) should be considered or trialed with intraoperative CLE.

## Data Availability Statement

The raw data supporting the conclusions of this article will be made available by the authors, without undue reservation.

## Ethics Statement

The studies involving human participants were reviewed and approved by St. Joseph's Hospital and Medical Center Institutional Review Board for Human Research. The patients and participants provided written informed consent to participate in this study.

## Author Contributions

EB, PN, and MP: study planning and coordination. EB and IA: acquisition of confocal images. EB, NO, XZ, and IA: processing and organizing of the data and confocal images. EB, XZ, IA, JE, PN, and MP: assessment of confocal images. EB, NO, and MP: writing the draft. MP: final approval. All authors: review of the draft.

## Conflict of Interest

The authors declare that the research was conducted in the absence of any commercial or financial relationships that could be construed as a potential conflict of interest.

## Abbreviations

CLE: confocal laser endomicroscopy
FET-PET: ^18^F-fluoroethyl-l-tyrosine–positron emission tomography
FLAIR: fluid-attenuated inversion recovery
FNa: fluorescein sodium
HGG: high-grade gliomas
IDH: isocitrate dehydrogenase
LGG: low-grade gliomas
MRI: magnetic resonance imaging
WHO: World Health Organization
5-ALA: 5-aminolevulinic acid.
