# Corrigendum: High-dose fluorescein reveals unusual confocal endomicroscope imaging of low-grade glioma

**DOI:** 10.3389/fneur.2025.1625940

**Published:** 2025-06-06

**Authors:** Evgenii Belykh, Naomi R. Onaka, Xiaochun Zhao, Irakliy Abramov, Jennifer M. Eschbacher, Peter Nakaji, Mark C. Preul

**Affiliations:** ^1^Department of Neurosurgery, The Loyal and Edith Davis Neurosurgical Research Laboratory, St. Joseph's Hospital and Medical Center, Barrow Neurological Institute, Phoenix, AZ, United States; ^2^Department of Neuropathology, Barrow Neurological Institute, St. Joseph's Hospital and Medical Center, Phoenix, AZ, United States

**Keywords:** confocal laser endomicroscopy, fluorescein sodium, fluorescence-guided surgery, low-grade glioma, nonenhancing glioma, oligodendroglioma

In the published article, there was an error in the Figure 2F Copyright statement. The statement was incorrectly written as:

“Figure 2F is adapted from Lv D, Hu Z, Lu L, Lu H, Xu X. Three-dimensional cell culture: A powerful tool in tumor research and drug discovery. Oncol Lett. 2017;14(6):6999-7010. Copyright of Institute of Pathology and Southwest Cancer Center and made available under Creative Commons Attribution 3.0 License (https://creativecommons.org/licenses/by/3.0).”

The corrected statement is:

“Figure 2F is adapted with permission from Lv et al. (22), CC-BY 4.0.”

The authors apologize for this error and state that this does not change the scientific conclusions of the article in any way. The original article has been updated.
